# The Novel H10N3 Avian Influenza Virus Triggers Lethal Cytokine Storm by Activating Multiple Forms of Programmed Cell Death in Mammalian Lungs

**DOI:** 10.3390/ijms26051977

**Published:** 2025-02-25

**Authors:** Xin Wang, Xiyue Wang, Xiaojuan Hao, Ruyi Gao, Xiaolong Lu, Wenhao Yang, Yu Chen, Jiao Hu, Min Gu, Xiaowen Liu, Shunlin Hu, Kaituo Liu, Xiaoquan Wang, Xiufan Liu

**Affiliations:** 1 Key Laboratory of Avian Bioproducts Development, College of Veterinary Medicine, Yangzhou University, Yangzhou 225009, China; wangxin_yzu@163.com (X.W.); bioyusy@outlook.com (X.W.); 18719560177@163.com (X.H.); 007622@yzu.edu.cn (R.G.); 008328@yzu.edu.cn (X.L.); 008465@yzu.edu.cn (W.Y.); biochenyu@hotmail.com (Y.C.); hujiaohot@163.com (J.H.); gumin@yzu.edu.cn (M.G.); xwliu@yzu.edu.cn (X.L.); slhu@yzu.edu.cn (S.H.); xfliu@yzu.edu.cn (X.L.); 2 Jiangsu Co-Innovation Center for Prevention and Control of Important Animal Infectious Diseases and Zoonosis, Yangzhou University, Yangzhou 225009, China; 3 Jiangsu Key Laboratory of Zoonosis, Yangzhou University, Yangzhou 225009, China; 4 Joint International Research Laboratory of Agriculture and Agri-Product Safety, The Ministry of Education of China, Yangzhou University, Yangzhou 225009, China

**Keywords:** H10N3 AIV, cytokine storm, programmed cell death, pathogenic mechanism

## Abstract

The novel H10N3 avian influenza virus (AIV) has infected four individuals since 2021 and caused severe respiratory damage, posing a significant threat to public health. However, its pathogenic mechanisms remain poorly understood. Our findings revealed that H10N3 infection induces severe lung damage and causes death in mice, even at low doses. The elevated levels of multiple pro-inflammatory factors in the bronchoalveolar lavage fluid were significantly increased during infection, displaying hallmarks of a cytokine storm. Transcriptome sequencing further revealed systematic activation of inflammation-related pathways, predicting that viral infection induces multiple forms of programmed cell death, including apoptosis, pyroptosis, and necroptosis. Protein-level validation showed that the activation of key cell death markers, including Caspase-3, GSDMD, and MLKL, significantly increased as the infection progressed, with their dynamic changes correlating strongly with the expression pattern of viral proteins. This study elucidates the central role of the synergistic effect between the cytokine storm and multiple cell death pathways in H10N3 pathogenesis. These findings not only advance our understanding of the pathogenic mechanisms of AIVs but also provide a critical theoretical basis for the development of targeted therapeutic strategies.

## 1. Introduction

Avian influenza virus (AIV) is one of the most significant pathogens that continuously threaten global public health security [1,2,3,4,5,6,7,8,9]. Based on the antigenicity of hemagglutinin (HA) and neuraminidase (NA) proteins on the viral envelope, AIV is currently classified into 16 HA subtypes (H1–H16) and 9 NA subtypes (N1–N9) [10]. In recent years, multiple AIV subtypes have frequently crossed species barriers to infect mammals and humans, posing significant threats to global public health [11,12,13,14,15]. Among these subtypes, the H10N3 virus was first reported to infect humans in 2021 in China [15]. To date, four human cases have been documented globally, with the most recent case reported in Guangxi Zhuang Autonomous Region, China, in December 2024 [15,16,17]. Our previous studies have demonstrated that the novel H10N3 virus exhibits high affinity for human-type receptors, high pathogenicity in mice, and efficient transmission between mammals through respiratory droplets [5,18,19]. These findings suggest that the novel H10N3 virus has the potential to evolve into a pandemic pathogen; however, its pathogenic mechanisms remain poorly understood.

In mammals, AIVs primarily target the lungs, where severe tissue damage leads to respiratory failure and mortality. The inflammatory cytokine storm induced by AIV infection, rather than direct viral damage, is the predominant factor contributing to lung injury [20,21,22,23]. Several studies have demonstrated that pro-inflammatory cytokines, including IL-1, IL-6, interferon-γ (IFN-γ), and tumor necrosis factor (TNF), are excessively released during AIV infection, leading to tissue damage and multiple organ failure [24,25,26,27]. However, the underlying mechanisms triggering these cytokine storms remain incompletely understood. Furthermore, virus-induced cell death pathways, including apoptosis, pyroptosis, and necroptosis, not only result from direct viral infection but also amplify inflammatory responses through the release of danger-associated molecular patterns (DAMPs), thereby accelerating disease progression [28].

This study aims to elucidate the pulmonary pathological damage induced by novel H10N3 infection and its underlying molecular mechanisms. We employed multiple experimental approaches, including in vivo infection models, cytokine profiling of bronchoalveolar lavage fluid, whole-transcriptome sequencing, and dynamic monitoring of cell death markers. Through these methodologies, we investigated the mechanisms of cytokine storm and multiple forms of cell death in H10N3-induced severe pulmonary pathology. Our findings advance the understanding of H10N3 pathogenesis and provide potential targets for therapeutic intervention.

## 2. Results

### 2.1. H10N3 Infection Causes Mouse Mortality and Severe Pulmonary Damage

**Figure 1:**
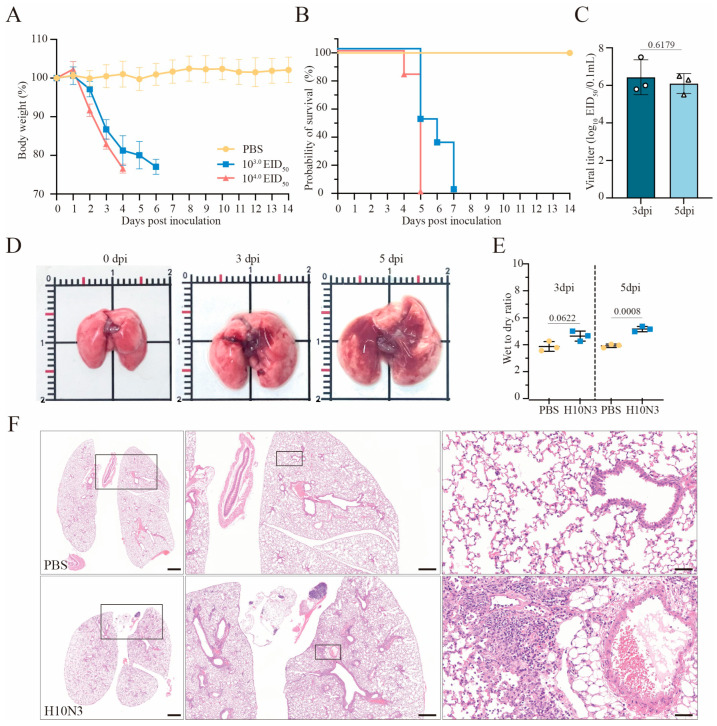
H10N3 virus infection causes mortality and severe pulmonary damage in mice. Groups of C57BL/6J mice (*n* = 6 per group, six weeks old, female) were intranasally inoculated with H10N3 virus at doses of 10^3.0^ and 10^4.0^ EID_50_, or PBS as mock-infected controls; body weight (**A**) and survival (**B**) were monitored daily for 14 dpi. Mice were humanely sacrificed when losing ≥25% of their initial body weight. (**C**) Mice infected with 10^3.0^ EID_50_ were euthanized at 3 and 5 dpi. Infectious viral titers in lung homogenates were quantified by EID_50_ assays in embryonated chicken eggs. (**D**) Representative gross pathological changes in mouse lungs at 0, 3, and 5 dpi following infection with 10^3.0^ EID_50_ of H10N3 virus. (**E**) Wet-to-dry weight ratios of lung tissues from PBS-treated and H10N3-infected mice at 3 and 5 dpi. (**F**) Representative H&E-stained lung sections at 3 dpi. Overview of whole lung sections (**left**, scale bar = 1000 μm), intermediate magnification of boxed regions showing tissue architecture (**middle**, scale bar = 500 μm), and high-magnification views highlighting inflammatory infiltrates, tissue damage, and hemorrhage (**right**, scale bar = 50 μm). Data are expressed as mean ± standard deviation (SD). Statistical analyses were performed using GraphPad Prism (v10.4.1), and *p*-values were calculated with a two-tailed unpaired Student’s *t*-test.

Six-week-old C57BL/6J mice were intranasally infected with the H10N3 virus at doses of 10^3.0^ or 10^4.0^ 50% Egg Infective Dose (EID_50_), or phosphate-buffered saline (PBS) as mock-infected controls. Infected mice exhibited significant weight loss starting on day 2 post-infection (Figure 1A). In the 10^3.0^ EID_50_ dose group, all mice died by day 7 post-infection, and in the 10^4.0^ EID_50_ dose group, mortality occurred earlier, by day 5 post-infection (Figure 1B). These results indicate that H10N3 infection severely compromised the overall health status of mice and significantly increased their mortality risk.

Lung tissues from mice inoculated with 10^3.0^ EID_50_ of H10N3 virus were collected at 3 and 5 days post-infection (dpi) for viral titer determination. The results showed that the H10N3 virus established an infection and efficiently replicated in lung tissues, thereby providing evidence for its high pathogenicity (Figure 1C).

Gross examination revealed significant pathological changes in the lung tissues of H10N3-infected mice. The infected lungs exhibited swelling and dark red areas of consolidation, suggesting virus-induced vascular damage and inflammatory cell infiltration (Figure 1D). Quantitative analysis of lung wet-to-dry weight ratios revealed evident edema in mouse lung tissues at 5 dpi (*p* = 0.0008), further evidence of significant pulmonary vascular damage (Figure 1E).

Histopathological analysis further revealed extensive lung damage caused by H10N3 virus infection. Hematoxylin–eosin (H&E) staining revealed extensive inflammatory cell infiltration in the alveolar spaces, along with notable thickening of alveolar walls and destruction of the peribronchial tissue structure (Figure 1F). These pathological changes indicate that H10N3 virus infection induced severe inflammatory responses and tissue damage.

In conclusion, these results indicate the comprehensive damage caused by H10N3 virus infection in mice through multidimensional experimental evidence, including significant weight loss, markedly shortened survival time, sustained viral replication in lung tissue, and resultant extensive pulmonary pathology.

### 2.2. H10N3 Infection Induces Significant Cytokine Storm

**Figure 2:**
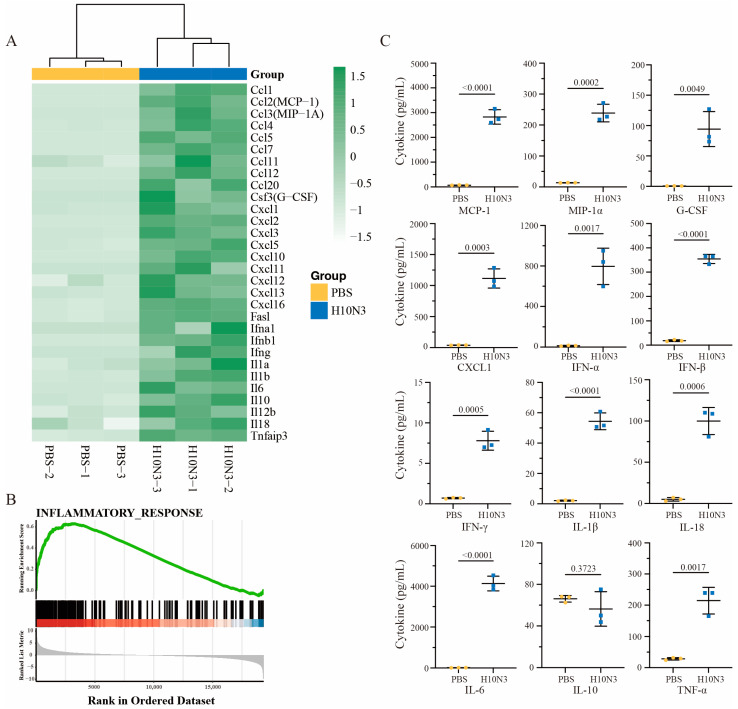
H10N3 virus infection induces inflammatory cytokine response in mice. Groups of C57BL/6J mice (*n* = 3) were inoculated with either PBS as a control or H10N3 virus at a dose of 10^3.0^ EID_50_. Samples were collected at 3 dpi for transcriptome analysis, and BALF was collected for cytokine measurement. (**A**) Heatmap showing hierarchical clustering of differentially expressed inflammatory genes (false discovery rate, FDR < 0.05; |log_2_ fold change, FC| > 1). The color scale represents row Z-score normalized expression values of fragments per kilobase of transcript per million mapped reads (FPKM) ranging from −1.5 (white, low expression) to 1.5 (green, high expression). (**B**) GSEA of the inflammatory response pathway showing significant enrichment in H10N3-infected samples. Top panel shows the enrichment score profile (green line) with a peak normalized enrichment score (NES) of approximately 0.6. Middle panel shows the positions of inflammatory response genes (black bars) in the ranked gene list. Below the black bars, a color bar (red to blue) indicates the direction and magnitude of gene expression changes, with red representing upregulation and blue representing downregulation in the H10N3-infected group compared to the control. Bottom panel indicates the ranked list metric (gray) showing the distribution of expression differences between PBS and H10N3 groups. (**C**) Protein levels of inflammatory cytokines in BALF quantified by Luminex multiplex assay. Data are presented as mean ± SD. *p*-values were calculated using a two-tailed unpaired Student’s *t*-test.

We conducted an in-depth investigation of immune response mechanisms following H10N3 virus infection. Mice were infected with 10^3.0^ EID_50_, and lung tissues were collected for transcriptome sequencing analysis at 3 dpi. Transcriptome analysis revealed significant changes in inflammation-related gene expression profiles between H10N3-infected and control groups. Heatmap analysis revealed significantly elevated expression of chemokine gene families (including CCL2/MCP-1, CCL3/MIP-1A, CCL4, CCL5, and CCL7) and cytokine genes (IL-6, IL-10, IL-18, and TNF). Hierarchical clustering analysis demonstrated distinct gene expression patterns between infected and control groups (Figure 2A).

Gene set enrichment analysis (GSEA) further confirmed significant activation of inflammatory response pathways. Enrichment plots showed significant positive enrichment of inflammation-related gene sets among all ranked genes, with a normalized enrichment score (NES) peaking at approximately 0.6. Leading edge analysis revealed a large cluster of inflammation-related genes concentrated at the front of the gene ranking, indicating their crucial role in H10N3 infection response. The distribution plot of gene expression changes confirmed significant upregulation of numerous genes in the infected group (Figure 2B).

Protein-level validation of bronchoalveolar lavage fluid (BALF) from mice at 3 dpi showed high consistency with transcriptome data. Key inflammatory cytokine levels were significantly elevated: IFN-β increased to approximately 350 pg/mL (*p* < 0.0001), IL-6 to approximately 4000 pg/mL (*p* < 0.0001), and TNF-α to approximately 200 pg/mL (*p* = 0.0017). Additionally, chemokines MCP-1 and MIP-1α increased to approximately 3000 pg/mL (*p* < 0.0001) and 250 pg/mL (*p* = 0.0002), respectively, further confirming chemokine pathway activation (Figure 2C).

Our multiomics analysis revealed the molecular mechanisms underlying H10N3-infection-induced cytokine storm. The consistent changes observed at both transcriptome and proteome levels indicate that viral infection triggers a systematic inflammatory response. Notably, the significant upregulation of chemokines likely promotes inflammatory cell recruitment, thereby exacerbating local inflammatory responses. This systematic inflammatory response likely represents the molecular basis for pulmonary injury. This finding provides important clues for understanding the pathogenic mechanisms of H10N3 while also offering new targets for potential therapeutic strategy development.

### 2.3. H10N3 Infection Activates Multiple Forms of Cell Death

Having established that H10N3 infection triggers a significant cytokine storm, we next investigated whether this inflammatory response was accompanied by changes in cell death pathways. Transcriptome analysis revealed that alongside cytokine upregulation, H10N3 infection induced substantial changes in multiple signaling pathways, particularly those involved in programmed cell death.

**Figure 3:**
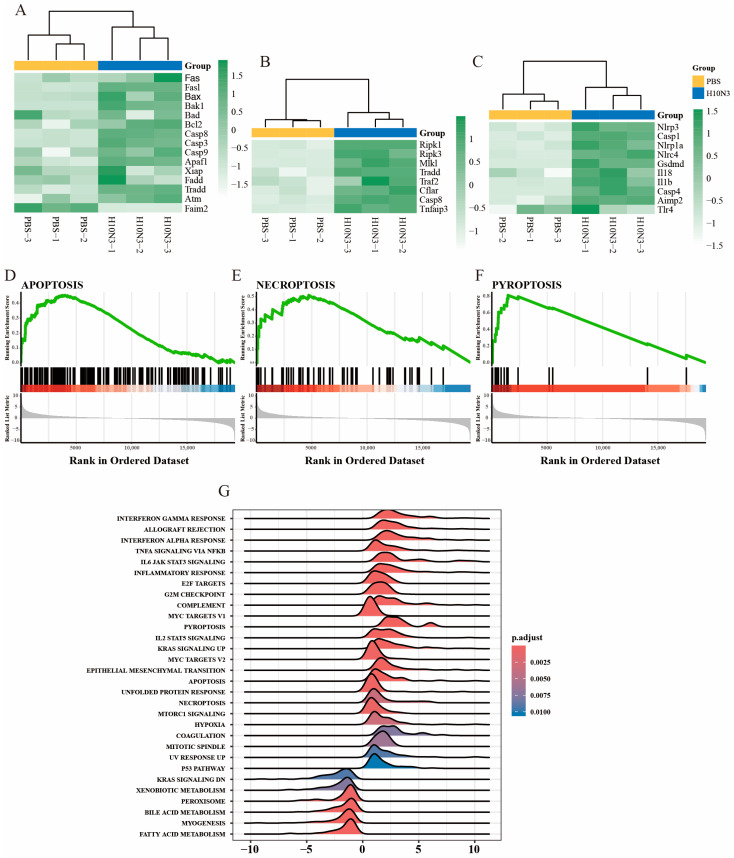
H10N3 virus infection activates multiple forms of cell death. Transcriptome analysis was performed on lung tissues from mice intranasally administered PBS (control group) or 10^3.0^ EID_50_ of H10N3 AIV (infected group), with tissues harvested at 3 dpi. (**A**–**C**) Heatmaps showing hierarchical clustering of differentially expressed genes (FDR < 0.05, |log_2_FC| > 1) involved in (**A**) apoptosis, (**B**) necroptosis, and (**C**) pyroptosis pathways. Color scales represent Z-score normalized expression values of FPKM. (**D**–**F**) GSEA showing significant enrichment of (**D**) apoptosis, (**E**) necroptosis, and (**F**) pyroptosis pathways in H10N3-infected samples. (**G**) Ridge plot showing significantly enriched pathways in H10N3-infected samples. The *x*-axis represents normalized enrichment scores, and the color gradient represents Benjamini–Hochberg adjusted *p*-values. Positive scores (**right**) represent upregulation, while negative scores (**left**) represent downregulation of pathways.

Among apoptosis-related genes, both extrinsic pathway molecules (Fas and FasL) and intrinsic pathway genes (Bax and Bad) were significantly upregulated. Downstream effector molecules, including Casp8 (extrinsic pathway), Casp9 (intrinsic pathway), and Casp3 (common pathway), exhibited coordinated activation patterns. Notably, the anti-apoptotic gene Faim2 was significantly suppressed, suggesting that H10N3 infection may enhance the apoptotic process by inhibiting anti-apoptotic factors (Figure 3A).

In the necroptosis pathway, we observed a significant increase in the transcription levels of key regulatory molecules RIPK1, RIPK3, and MLKL. Additionally, upstream regulators Tradd, Traf2, and the adaptor molecule Cflar also showed significant upregulation, indicating that the signaling pathway of necroptotic cell death was systematically activated (Figure 3B). At the same time, in the pyroptosis pathway, key components of the inflammasome, such as Nlrp3, Nlrp1, and Nlrc4, showed significant increases in expression, accompanied by the coordinated activation of downstream effector molecules Casp1, Casp4, and the effector molecule GSDMD. The expression levels of pro-inflammatory cytokines Il1b and Il18 were also significantly increased, indicating significant enhancement of pyroptosis-mediated inflammatory response (Figure 3C).

To further validate these findings, we performed gene set enrichment analysis. The results indicated that the three cell death pathways—apoptosis (Figure 3D), necroptosis (Figure 3E), and pyroptosis (Figure 3F)—all showed significant positive enrichment patterns, with enrichment scores reaching statistically significant levels. Broader pathway enrichment analysis further revealed that, in addition to cell-death-related pathways, multiple immune-response-related pathways were also significantly activated, including the interferon-γ response, interferon-α response, TNF-α via the NF-κB signaling pathway, and the IL6-JAK-STAT signaling pathway.

Moreover, immune-related pathways such as the inflammatory response and the complement system, as well as cell cycle regulation pathways like the G2M checkpoint and mitotic spindle, also showed significant enrichment. Additionally, metabolic pathways such as fatty acid metabolism, myogenesis, and peroxisome were also significantly affected (Figure 3G).

These results suggest that H10N3 infection mediates the clearance of infected cells through concurrent activation of apoptosis, necroptosis, and pyroptosis pathways, accompanied by a robust immune–inflammatory response. Furthermore, H10N3 infection significantly impacts the host cell’s basic physiological processes, including cell cycle regulation and metabolic reprogramming. These coordinated cellular responses reveal the complex pathogenic mechanisms of H10N3 virus infection and provide a molecular framework for understanding virus–host interactions.

### 2.4. Activation Validation of Cell Death Marker Proteins

To further clarify the forms of programmed cell death induced by H10N3 subtype AIV infection and its molecular mechanisms, mice were infected with a dose of 10^3.0^ EID_50_. The expression and activation status of key programmed cell death marker proteins in lung tissue were systematically analyzed by Western blot on days 1, 3, and 5 post-infections.

**Figure 4:**
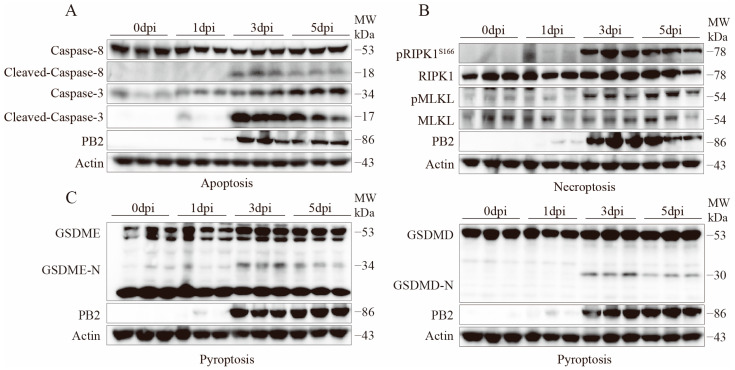
H10N3 infection activates multiple cell death pathways in mouse lung tissue. Mice were infected with H10N3 virus at 10^3.0^ EID_50_, and lung tissues were collected at 0, 1, 3, and 5 dpi. (**A**) Western blot analysis of apoptosis markers including total and cleaved forms of Caspase-8 and Caspase-3. (**B**) Western blot analysis of necroptosis markers including phosphorylated receptor-interacting serine/threonine-protein kinase 1 at Serine 166 (pRIPK1^S166^), total RIPK1, phosphorylated mixed lineage kinase domain-like protein at Serine 345 (pMLKL^S345^), and total MLKL. (**C**) Western blot analysis of pyroptosis markers including full-length and N-terminal fragments of gasdermin E (GSDME) and gasdermin D (GSDMD).

Multiple cell death pathways were activated during H10N3 infection. In apoptosis, cleaved Caspase-8 and Caspase-3 levels significantly increased by 3 dpi (Figure 4A). Necroptosis markers pRIPK1^S166^ and pMLKL^S345^ showed progressive phosphorylation from 3 dpi onward, peaking at 3–5 dpi (Figure 4B). Simultaneously, pyroptosis executors GSDME-N and GSDMD-N fragments were elevated at 3–5 dpi, indicating gasdermin-mediated pore formation (Figure 4C).

Furthermore, the expression of the viral protein PB2 correlated with the activation of various cell death markers, showing a significant increase starting from 3 dpi, suggesting that viral replication may be a key factor triggering the activation of multiple cell death pathways.

These results demonstrate that H10N3 virus infection induces concurrent activation of multiple programmed cell death pathways, including apoptosis, necroptosis, and pyroptosis, in mouse lung tissue. The activation of these pathways exhibits a time-dependent pattern. The coordinated activation of multiple cell death pathways may represent a crucial molecular mechanism underlying the pulmonary pathological damage associated with H10N3 infection.

## 3. Discussion

In this study, we investigated the molecular mechanisms underlying H10N3-induced mammalian lung injury. Our findings demonstrate that H10N3 infection simultaneously activates multiple forms of programmed cell death, including apoptosis, necroptosis, and pyroptosis, resulting in massive cellular destruction in lung tissue. The dying cells release substantial amounts of virus particles and DAMPs, triggering inflammatory cascades and cytokine storms, ultimately resulting in severe lung damage, respiratory failure, and host mortality.

Compared to other respiratory viruses, H10N3 exhibits multifaceted effects in the induction of cell death. For instance, respiratory syncytial virus (RSV) infection primarily induces cell death through pyroptosis and necroptosis pathways [29]. Similarly, in severe acute respiratory syndrome coronavirus 2 (SARS-CoV-2) infection of human airway epithelial cells, single-cell and limiting dilution analyses revealed that necroptosis is the predominant cell death event in infected cells [30]. In contrast, H10N3’s ability to simultaneously activate multiple death pathways represents a crucial factor contributing to its distinctive pathogenicity, potentially through the synergistic effects of different cell death mechanisms and the subsequent amplification of inflammatory responses.

Viral infections can activate sensor proteins through two main pathways—extrinsic and intrinsic—thereby inducing programmed cell death [31]. The extrinsic pathway is predominantly mediated by inflammatory factors. Studies have demonstrated that viral infections activate the innate immune system, leading to the production of inflammatory factors such as TNF-α and IFN-β. These factors subsequently activate death receptors, including TNFR and IFNAR. These receptors form complexes with proteins such as RIPK1, RIPK3, and FADD through the RHIM domain, thereby regulating apoptosis and necroptosis, highlighting the critical role of the extrinsic pathway in inducing cell death [31,32]. In contrast, the intrinsic pathway is dependent on the viral replication process and is primarily mediated through interactions between the viral genome (vRNA), viral-encoded proteins, and sensor proteins [31].

This study demonstrated that, following H10N3 AIV infection in mice, viral PB2 protein expression reached its peak at 3 dpi. This peak coincided with enhanced activation of programmed cell death markers, indicating a potential relationship between viral replication and intrinsic-pathway-mediated cell death. Moreover, H10N3 infection elicited robust inflammatory responses, characterized by clinical manifestations of cytokine storm including pulmonary edema and respiratory distress, suggesting concurrent activation of the extrinsic death pathway [33]. Notably, different types of programmed cell death release specific DAMPs, which can induce distinct inflammatory responses. In particular, IL-1β and IL-18 released during pyroptosis primarily promote neutrophil recruitment and activation, while DAMPs released during necroptosis mainly activate macrophages through TLR signaling pathways [34,35]. To elucidate the molecular mechanisms linking programmed cell death and inflammatory responses, we performed transcriptome sequencing analysis. The results showed significant upregulation of genes encoding pro-inflammatory factors, including IL-6, TNF-α, and IL-1β. Subsequent protein-level validation further confirmed the substantial production of these key inflammatory factors. The widespread release of these inflammatory cytokines indicates that cell death triggered an extensive cytokine storm, which not only directly damaged tissues but also led to massive infiltration and activation of immune cells in the tissue microenvironment, establishing a positive feedback loop of inflammatory responses.

Gene set enrichment analysis further revealed synergistic activation between programmed-cell-death-related pathways and inflammatory signaling pathways, particularly the co-upregulation of key signaling pathways such as TNF-α-NF-κB and IL6-JAK-STAT. Enhanced expression of key regulatory molecules RIPK1, RIPK3, and CASP8 indicates that virus-induced cell death amplifies inflammatory responses via diverse mechanisms. Among these, RIPK1 not only initiates necroptosis but also mediates the transcription of pro-inflammatory factors through regulation of the NF-κB pathway, thereby serving as a molecular signaling bridge between cell death and inflammation [36,37].

While programmed cell death serves as a critical host defense against virus-infected cells, its dysregulation can lead to extensive tissue damage. Recent evidence indicates that excessive cell death contributes more significantly to disease progression than direct viral cytopathic effects [38]. In influenza virus infections, the pathogenesis is primarily driven by excessive inflammation, widespread pulmonary epithelial cell death, and subsequent acute lung injury [39]. In TNF-α and IL-1 receptor knockout mice, even when infected with highly pathogenic H5N1 AIVs, the severity of lung inflammation was significantly reduced, and survival time was notably prolonged [40]. This finding clearly demonstrates the positive role of inhibiting cytokine storms in mitigating disease progression. A similar phenomenon is observed in other viral pneumonias. For instance, in COVID-19, children often exhibit higher viral loads but rarely develop severe pneumonia [41]. Moreover, viral loads in respiratory samples from SARS-CoV- and SARS-CoV-2-infected patients peak before clinical symptom onset, with disease severity correlating primarily with immune dysregulation and cytokine storm intensity [42]. These observations suggest that disease severity depends more on immunological dysregulation than viral burden.

From a clinical perspective, H10N3 infection activates both intrinsic and extrinsic cell death pathways synergistically, triggering cytokine storm and potentially contributing to poor disease outcomes. Excessive inflammatory cytokine release causes progressive lung tissue damage, leading to respiratory failure and mortality. These findings suggest that targeting both programmed cell death pathways and inflammatory responses could provide effective therapeutic interventions for H10N3 infection. Necroptosis inhibitors have shown promising therapeutic efficacy in various viral diseases [43]. Thus, further investigation into the regulatory mechanisms of these programmed cell death pathways and the development of corresponding targeted therapeutic strategies may provide new breakthroughs in controlling host damage caused by AIV infections.

Despite these advances, several key questions remain to be addressed. Although we have revealed the activation characteristics of multiple cell death pathways, the interaction network and precise regulatory mechanisms between these pathways remain unclear. Specifically, does cross-regulation exist between different types of cell death? Do they cooperate to amplify the inflammatory response? These critical questions necessitate further investigation. Beyond its role in viral pathogenesis, cell death serves as a viral strategy to circumvent host immune surveillance [44,45]. Further investigation of these aspects will clarify the roles of programmed cell death in viral infections and facilitate the development of targeted antiviral therapies.

Through multiomics analysis, this study unveiled the intricate interactions between intrinsic and extrinsic programmed cell death pathways in modulating cytokine storms during H10N3 infection, establishing a novel framework for understanding AIV pathogenesis. Our findings demonstrate that the coordinated activation of multiple cell death pathways serves as a critical determinant of viral pathogenicity. These insights emphasize that effective therapeutic strategies must simultaneously address both direct viral effects and death-signal-mediated cytokine storms. The findings not only advance our understanding of AIV prevention and treatment but also provide valuable mechanistic insights applicable to therapeutic strategy optimization in other viral pneumonias.

## 4. Materials and Methods

### 4.1. Virus

The novel H10N3 strain (A/chicken/Jiangsu/0110/2019) was isolated from chickens and maintained in our laboratory collection [19]. For propagation and amplification, the virus was cultured in 9-day-old specific-pathogen-free (SPF) embryonated chicken eggs, then preserved at −80 °C for subsequent experiments.

### 4.2. Mouse Challenge Experiments

Female C57BL/6J mice (six weeks old) were obtained from the Yangzhou Experimental Animal Center (Yangzhou, Jiangsu Province, China). Following anesthesia, groups of mice (*n* = 6) received intranasal inoculation with 50 μL of H10N3 virus at two different concentrations, 10^3.0^ and 10^4.0^ EID_50_ in sterile PBS, respectively. The mice were monitored for weight changes and survival over 14 days. Animals were humanely euthanized if they experienced body weight loss ≥25% of their initial weight.

To assess viral replication in the respiratory tract, groups of three mice were administered 10^3.0^ EID_50_ of virus intranasally and sacrificed at 3 and 5 dpi. Their lungs were collected and processed in 1.0 mL PBS to measure viral loads using the EID_50_ method.

For lung edema assessment, whole lungs were harvested and weighed immediately (wet weight), then dried at 60 °C for 60 h before obtaining the dry weight. The wet/dry ratios were determined at both 3 and 5 dpi. For histological examination, lung tissues were harvested at 3 dpi and preserved in 4% paraformaldehyde solution, embedded in paraffin, and cut into 4 μm sections. The sections were stained with hematoxylin–eosin (H&E) assays.

### 4.3. RNA Detection, Library Construction, and Sequencing

Groups of mice (*n* = 3) were anesthetized and given intranasal administration of either 50 μL H10N3 virus (10^3.0^ EID_50_) in PBS or PBS alone (control group). At 3 dpi, the mice were euthanized in an ultra-clean environment and their lungs were harvested. The lung specimens were flash-frozen in liquid nitrogen for 10 min in 2 mL cryotubes, then transferred to −80 °C storage. These samples were subsequently processed at Shanghai Biotechnology Corporation (Shanghai, China) for RNA sequencing analysis.

Total RNA was extracted from three biological replicates using TRIzol reagent (Thermo Fisher Scientific, Waltham, MA, USA), with RNA integrity numbers (RINs) > 8.0 verified by Agilent 4200 Bioanalyzer analysis (Agilent Technologies, Santa Clara, CA, USA). Strand-specific libraries were prepared with the VAHTS Universal V6 RNA-seq Kit (Vazyme Biotech, Nanjing, Jiangsu, China) using poly(A) selection. After adapter ligation and PCR amplification (12 cycles), libraries were quantified using Qubit 4.0 Fluorometer (Thermo Fisher Scientific, Waltham, MA, USA) and size-selected for 300–500 bp insert fragments.

Paired-end sequencing (2 × 150 bp) was performed on an Illumina NovaSeq 6000 platform (Illumina, San Diego, CA, USA), generating 40 million paired-end reads per sample. Raw FASTQ files were processed with Seqtk (v1.3, GitHub: https://github.com/lh3/seqtk, accessed on 21 February 2025) for quality trimming. Clean reads were subsequently aligned to the mouse reference genome GRCm39 using HISAT2 (v2.0.4, Kim Lab, Johns Hopkins University, Baltimore, MD, USA) [46]. SAM files were converted to sorted BAM files using SAMtools (v1.11, Wellcome Sanger Institute, Hinxton, UK).

TMM normalization, implemented using edgeR (v3.36.0, Bioconductor Project, Fred Hutchinson Cancer Research Center, Seattle, WA, USA), was applied to correct for systematic biases in sequencing depth and library composition [47,48,49,50]. For visualization, FPKM values were calculated to account for gene length differences using the following formula:

$$FPKM=\frac{Exonic\;fragments}{\left[Gene\;length\left(kb\right)\times Total\;mapped\;fragments(million)\right]}$$

Differential expression analysis employed edgeR’s quasi-likelihood F-test with Benjamini–Hochberg adjusted *p*-values (FDR < 0.05) and |log2(fold change)| > 1 [51]. Expression values were z-score-normalized per gene:

$$Z_{ij}=\frac{{FPKM}_{ij}-\mu_{i}}{\sigma_{i}}$$

where μ_i_ and σ_i_ represent the mean and standard deviation of gene i across all samples.

### 4.4. Gene Set Enrichment Analysis (GSEA)

Pathway enrichment analysis was performed using the Hallmark gene sets from Molecular Signatures Database (MSigDB, v2024.1.Mm; Broad Institute, Cambridge, MA, USA) to evaluate the representation of genes in specific biological processes [52]. The GSEA Java program (v4.1; Broad Institute, Cambridge, MA, USA) was employed to analyze pathway enrichment patterns [53], with particular focus on pathways associated with apoptosis, necroptosis, pyroptosis, and inflammatory cytokine responses after viral infection. Following GSEA guidelines, pathways were considered significantly enriched when their false discovery rate (FDR) was below 25%.

### 4.5. Assessment of Inflammatory Mediators in Bronchoalveolar Lavage Fluid

At 3 dpi, bronchoalveolar lavage fluid samples were obtained from both PBS control and H10N3-infected mice. Cytokine and chemokine levels were measured using a Bio-Plex MAGPIX System (Bio-Rad Laboratories, Hercules, CA, USA) with Bio-Plex Pro Mouse Cytokine Grpl Panel 12-plex kit (Wayen Biotechnologies, Shanghai, China) according to the manufacturer’s protocols. The analysis procedure involved incubating the sample supernatants with microbead-embedded 96-well plates for 1 h, followed by a 30 min detection antibody incubation step. Finally, streptavidin-PE (Thermo Fisher Scientific, Waltham, MA, USA) was introduced to each well for 10 min before measurement.

### 4.6. Antibodies

Antibodies for mMLKL (phospho-S345) (#37333), MLKL (#37705), mRIPK1 (phospho-S166) (#53286), RIPK1 (#3493), mouse Cleaved-Caspase-8 (Asp387) (#8592), Caspase-8 (#4790), Cleaved-Caspase-3 (#9664), and Caspase-3 (#14220) were purchased from Cell Signaling Technology (Danvers, MA, USA). Anti-GSDMD antibody (#ab209845) and anti-GSDME antibody (#ab215191) were purchased from Abcam (Cambridge, UK). Anti-influenza A virus PB2 (#GTX125926) was purchased from GeneTex (Irvine, CA, USA). β-Actin antibody (#sc-47778) was purchased from Santa Cruz Biotechnology (Dallas, TX, USA).

### 4.7. Western Blot

For analysis of cell-death-related protein activation, lung tissues were harvested and homogenized in 1.0 mL RIPA lysis buffer (Beyotime Biotechnology, Shanghai, China; #P0013B). Following sonication, the homogenates were centrifuged at 12,000× *g* for 15 min at 4 °C. The protein concentration in the collected supernatants was quantified using a BCA protein assay kit (Beyotime Biotechnology; #P0012). The samples were then combined with 5X loading buffer (Beyotime Biotechnology; #P0015L), heated at 95 °C for 5 min, and equal protein amounts were separated by electrophoresis before transfer to PVDF membranes (Millipore, Burlington, MA, USA; #IPVH00010). Specific antibodies were used to detect target proteins via Western blot analysis.

### 4.8. Statistical Analysis

Data visualization and statistical evaluation were conducted using GraphPad Prism software (v10.4.1, GraphPad Software, San Diego, CA, USA). Results are expressed as mean ± SD. Comparisons between two groups were analyzed using a two-tailed unpaired Student’s *t*-test, with statistical significance set at *p* < 0.05.

## Data Availability

The complete dataset relevant to this study is presented herein. Additional data can be furnished upon request.
